# Open hardware microsecond dispersive transient absorption spectrometer for linear optical response

**DOI:** 10.1007/s43630-021-00127-6

**Published:** 2021-11-08

**Authors:** Christopher D. M. Hutchison, Susan Parker, Volha Chukhutsina, Jasper J. van Thor

**Affiliations:** 1 Department of Life Sciences, Imperial College London, London, SW7 2AZ UK; 2 QOLS Physics Group, Blackett Laboratory, Imperial College London, London, SW7 2BW UK

## Abstract

**Abstract:**

An open hardware design and implementation for a transient absorption spectrometer are presented that has microsecond time resolution and measures full difference spectra in the visible spectral region from 380 to 750 nm. The instrument has been designed to allow transient absorption spectroscopy measurements of either low or high quantum yield processes by combining intense sub-microsecond excitation flashes using a xenon lamp together with stroboscopic non-actinic white light probing using LED sources driven under high pulsed current from a capacitor bank. The instrument is sensitive to resolve 0.15 mOD flash-induced differences within 1000 measurements at 20 Hz repetition rate using an inexpensive CCD sensor with 200 μm pixel dimension, 40 K electrons full well capacity and a dynamic range of 1800. The excitation flash has 230 ns pulse duration and the 2 mJ flash energy allows spectral filtering while retaining high power density with focussing to generate mOD signals in the 10^–4^–10^–1^ ΔOD range. We present the full electronics design and construction of the flash and probe sources, the optics as well as the timing electronics and CCD spectrometer operation and modification for internal signal referencing. The performance characterisation and example measurements are demonstrated using microsecond TAS of Congo red dye, as an example of a low quantum yield photoreaction at 2% with up to 78% of molecules excited. The instrument is fully open hardware and combines inexpensive selection of commercial components, optics and electronics and allows linear response measurements of photoinduced reactions for the purpose of accurate global analysis of chemical dynamics.

**Graphical abstract:**

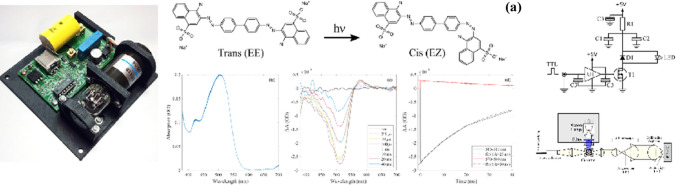

**Supplementary Information:**

The online version contains supplementary material available at 10.1007/s43630-021-00127-6.

## Introduction

The technique of Transient Absorption Spectroscopy (TAS) is well established, from the early work of George Porter and others who used flash sources for excitation and probing of chemical reactions [1–5] (Nobel Prize in Chemistry 1967). The instrumentation that is used for modern TAS applications typically employ nanosecond lasers together with single-wavelength probes to record kinetic traces for diagnostic frequencies of reactants and products [6, 7]. The use of white light probing and dispersive measurement of difference spectra in the full visible spectral region is less common, and often applies intense pulsed xenon sources that can be actinic (LP980 Edinburgh Instruments). Recording time-resolved spectral information is particularly powerful to separate heterogeneous reactions and to resolve multi-phasic decay and complex reaction pathways. For such analysis, the TAS data is analysed using Singular Value Decomposition (SVD), global fitting and target analysis techniques [8]. An alternative model-free method applies lifetime-density analysis that directly transforms the experimental data and may visualise the spectral dynamics in cases when lifetime distributions are evident in the data [9–12]. The choice to record full spectra rather than single wavelength traces has implications with regard to the reduced sensitivity of the difference absorption that can be detected. It is, therefore, important that the instrumentation combines both high enough sensitivity (typically ~ 0.15 mOD differences) together with large enough excitation power density to generate a sufficient dynamic range in the photoinduced difference measurements to allow the SVD and global analysis.

An alternative approach to collect nanosecond to sub-millisecond TAS is to extend conventional femtosecond transient absorption spectroscopy to include such longer pump-probe delays [13–15] with a secondary synchronised excitation laser source.

Another consideration and motivation to record microsecond TAS in addition to femtosecond TAS are to evaluate the linear excitation and product formation. With intense femtosecond visible laser pulses there can be many different types of non-linear optical processes that will not necessarily represent for example, those that occur in nature under weak continuous illumination of the sun. For instance, in photosynthesis research it is well established that exciton-exciton annihilation occurs in relatively small light-harvesting antennae (LHCII monomer) with pulse energies exceeding 8 μJ/cm^2^ and even lower for larger complexes [16–18]. Similar considerations apply to solid-state materials. For single chromophore systems, multi-photon processes can involve excited state absorption, photoionisation and stimulated emission pumping [19]. Even under very weak excitation conditions of single chromophore materials, the non-linear conditions cannot be avoided if the excitation pulse is short femtosecond duration. Namely, if the laser spectrum is narrower than the absorption line, then the four-wave mixing response will be included in the TAS signal. While weak excitation will suppress, but not remove, the ground state Impulsive Raman signal, the excited state coherence is purely displacement driven and per molecule independent of the power density [20–22]. Therefore, for many applications of femtosecond TAS, the analysis of the linear optical response is also of great interest.

Explicitly, to access the linear optical response the power density should be sufficiently low such that the Rabi frequency $\left| {\frac{{\left( {d_{ij} \times E} \right)}}{\hbar }} \right|$ is well below the detuning and dephasing parameter, described by the formula [23]:

1 $$\left| {\frac{{\left( {d_{ij} \times E} \right)}}{\hbar }} \right| \ll \left| {\omega_{ij} - \omega - i\gamma_{ij} } \right|,$$

where an electric field of strength, *E* and frequency, $\omega$ is used to drive a transition between state *i* and *j*, with a transition dipole moment *d*_*ij*_, central transition frequency, $\omega_{ij}$ and the damping constant, $\gamma_{ij}$. This condition is best met with microsecond, or sub-microsecond flashes, as even nanosecond laser pulses can drive a non-linear response.

Commercially available instrumentation for nanosecond TAS is costly to purchase and maintain, requires laser safety procedures and significant laboratory space. The excitation source is usually a Nd:YAG laser with either second harmonic generation (SHG), third-harmonic generation (THG), optical parametric oscillator (OPO) or dye laser for wavelength conversion providing typically ~ 7–10 ns excitation pulses. Furthermore, the ~ 10 ns time resolution is usually for single wavelength probes. To achieve the corresponding dispersive spectral measurement for such commercial systems, it is necessary to use a nanosecond gated image intensified CCD camera (ICCD) together with a bright and potentially actinic xenon pulsed probe (Edinburgh Instruments).

**Fig. 1:**
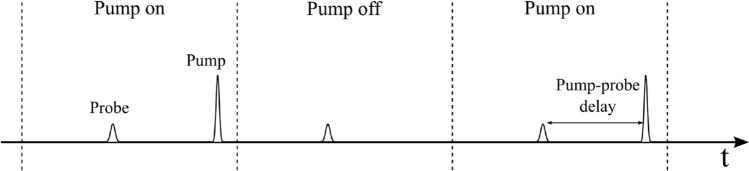
Pulse sequence diagram for the TAS setup. Dotted lines separate the pump on and pump off cycles which repeat every 100 ms

Here, we present and demonstrate the utility of an alternative TAS instrument that sacrifices some time resolution but ensures the linear response and records full spectra. In addition, it requires no pump laser, is very inexpensive to construct, compact and fully described with all schematics, specifications and design information. The instrument is based on an overdriven LED to provide microsecond white light probe pulses which are passed through a sample and measured using a fibre coupled spectrometer, while intense pump pulses are provided by a xenon flash lamp assembly. Probe pulses are delivered at 20 Hz (the cycle time of the spectrometer) while pump pulses at 10 Hz resulting in a “pump-on”/“pump-off” pulse sequence as shown in Fig. 1 which repeats every 100 ms. Longer pump-probe delays are possible with lower repetition rate. Spectra are read off the spectrometer board directly using a digital oscilloscope and averaged over repeated measurements. Comparison of the averaged pumped and un-pumped spectra allow light-induced spectral differences to be measured with better than mOD accuracy and with repeated measurements at different pump-probe delays the spectral kinetics can be resolved.

## Materials and methods

### System layout

**Fig. 2:**
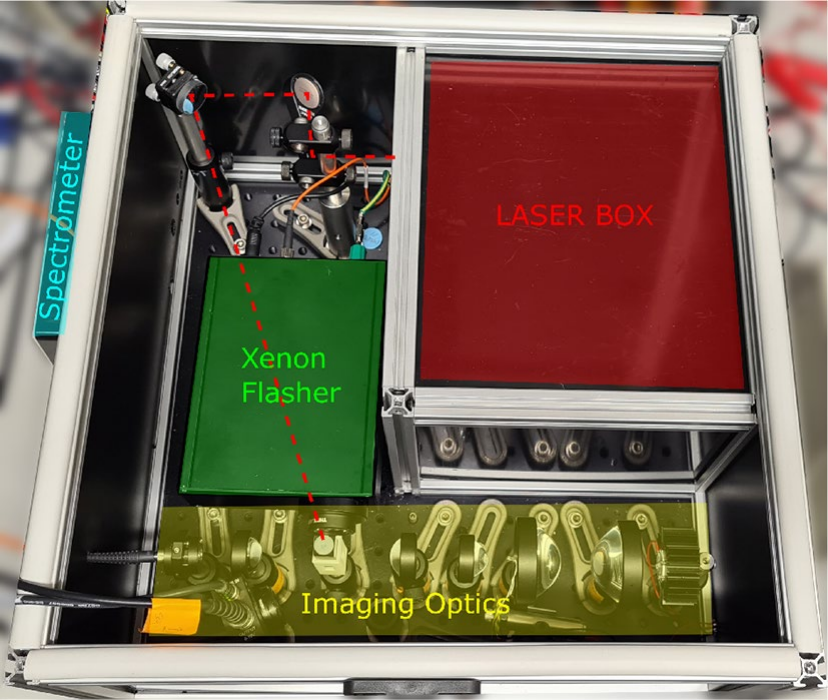
Layout of TAS setup showing the different parts of the main construction. The CW laser path (dashed red line) passes over the top of the xenon flasher and would be directed down through the top of the cuvette with a mirror (not pictured)

The system is constructed on a 450 × 450 mm optical breadboard as shown in Fig. 2 and fully enclosed. An over-driven white light LED provides microsecond probe pulses which are imaged through a three windowed, 3 mm path length quartz cuvette (Helma 105-251-15-40) and collected with a fibre coupled spectrometer. A xenon flasher positioned 90 degrees to the probe light provides intense (> 2 mJ) white light pump pulses that can be optically filtered to meet samples requirements. The construction includes a separate fully enclosed and shuttered “laser box” which allows high power Continuous Wave (CW) or Transistor–Transistor Logic (TTL) triggered laser to be used in conjunction with the transient absorption setup. The additional laser illumination allows stroboscopic measurements of reversible reactions such as cis/trans and trans/cis photoisomerisation, or the accumulation of meta-stable intermediates or photoequilibrium states. The CW laser is directed down through the top of the cuvette which is either left open or sealed with a cover slide and grease when working with a volatile sample. The laser box shutter is interlocked with the lid of the main box allowing the setup to operate a Class 1 laser system during normal operation, making it safe to use for users that are not experienced with high power lasers.

Samples that exhibit irrecoverable bleaches, do not thermally recover with the 50 ms cycle time of the setup or cannot be optically recycled can be continuously replenished through the use of a Flow-Through Cell (Helma 176.751-QS) and peristaltic pump. In this arrangement, it is still possible to pre-illuminate the sample “in line” by passing the tubing through the laser box and illuminating with an optical flow cell or directly through transparent tubing.

### Probe source

**Fig. 3:**
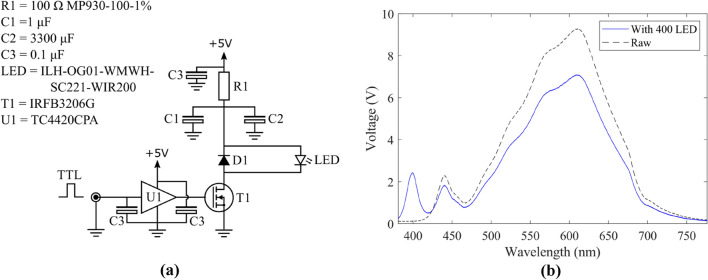
LED overdriving circuit diagram **a** used to drive white light LED probe and **b** the spectral profile of the probe as collected by the instrument with (solid) and without (dashed) the use of an augmenting 400 nm LED (described below)

To maximize the temporal resolution of the TAS it was critical to produce probe pulses with sufficient brightness to fill the dynamic range of the spectrometer while remaining as short as possible. This was done by overdriving a white light LED using a current pulse circuit (Fig. 3a). The circuit is based on an n-channel MOSFET (IRFB3206G) to control the discharge of a 1 μF and 3300 μF capacitors at 10 MHz bandwidth. The specific LED (OSLON Square PowerStar White LED (3000 K)) was chosen for its spectral profile (Fig. 3b), high power rating (2 W) and small source size to improve collimation and focusing. The relative brightness of the source was proportional to the length of the TTL trigger used, however, it was still possible to fill the full dynamic range of the spectrometer with ~ 1 μs pulses. 0.1 μF decoupling capacitors were used at several points in the circuit to help reduce electrical noise. 2 μs TTL pulses and 5 V potential was typically used and is non-destructive, the circuit does support the use of higher voltages (10–20 V) which consequently would allow further reduction of the light pulse duration while maintaining the same brightness according to the current and time duration product.

### Optical layout

**Fig. 4:**
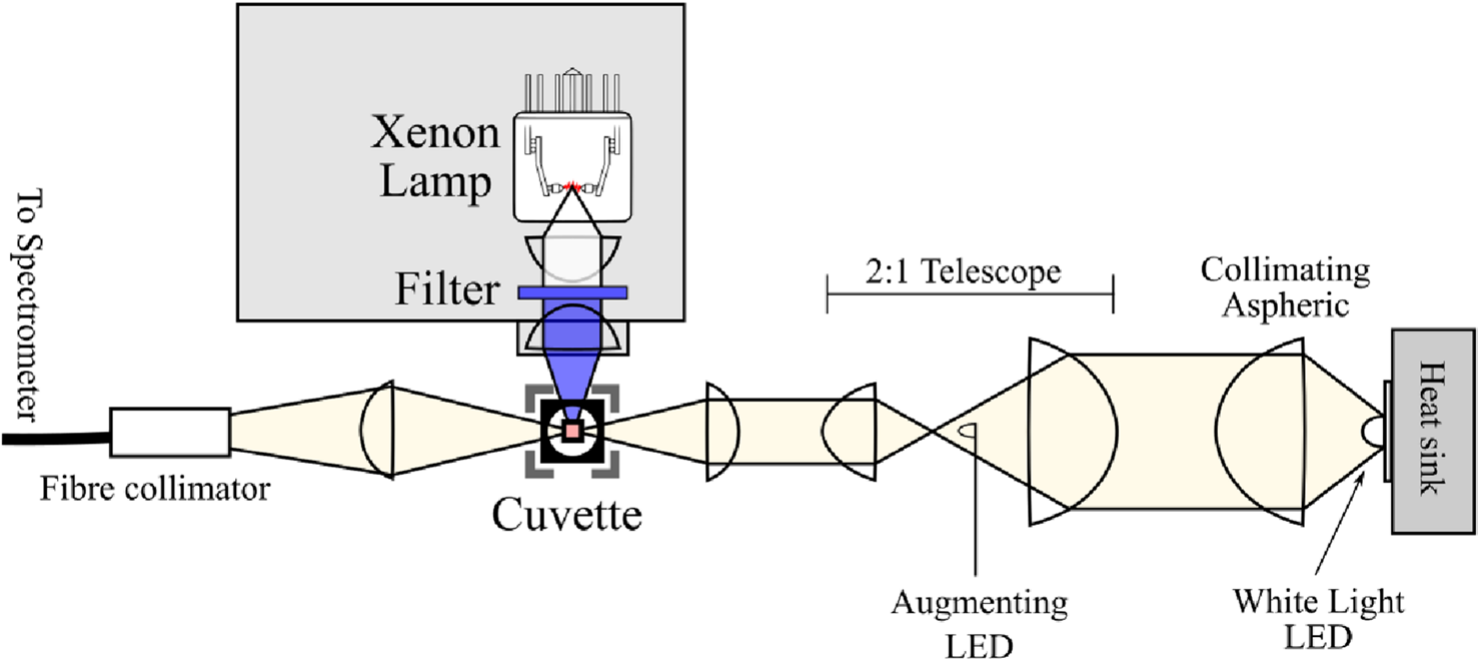
Optical layout of the millisecond TAS. Optical paths of the white light probe (yellow) as well as the full spectrum (white) and filtered (blue) pump light paths from the Xenon flasher are shown

The optical layout of the TAS is shown in Fig. 4. White light emission from the overdriven LED probe was collected and collimated using a 2″ *f* = 32 mm aspherical lens (Thorlabs ACL50832U). This beam was telescoped down to ~ 1″ using two more aspherics (Thorlabs ACL50832U, ACL25416U) and focused into the micro cuvette using a 50 mm lens. The combination of an additional 50 mm lens and fiber collimator is used to couple the output from the sample into the spectrometer. The white light LED spectra (Fig. 3b) extended from 420 to 750 nm, if additional spectral regions were required to probe outside of this range it was possible to introduce a small augmenting LED near to the focus of the telescope (Fig. 4), driven off the same current pulse as the white light, to add in missing wavelengths but at the cost of blocking a portion of white light. This was typically done with a 400 nm LED to result in a probe light spectra spanning 380–750 nm. We have similarly used an augmenting LED with a 735 nm central wavelength for making measurements of photosynthesis samples (not shown).

### Spectrometer

The fibre coupled spectrometer (B&W TEK, BRC100) utilises a linear array CCD sensor (ILX511 Sony), consisting of 2048, 14 μm wide pixels with vertical heights of 200 μm and has a full well depth capacity of 43.77 × 10^3^ e^−^, providing a single-shot upper limit to the Poisson related SNR of 209 [24]. The sensor has reported dynamic range (saturation voltage/read noise) of 1810 and a sensitivity of 780 V/lx.s [24]. The input slit is 50 μm. At 20 Hz line rate the integration time was set to the minimum value of 50 ms. Data acquisition at this full line rate is not supported by the onboard ADC and serial bus, therefore, was done by reading directly from the analogue board output using an external 16 bit (15 bit in dual channel mode) digital oscilloscope (Pico Technology, Picoscope 5244B). The analogue signal was oversampled at 2.5 MS per second (Sect. 3.9).

**Fig. 5:**
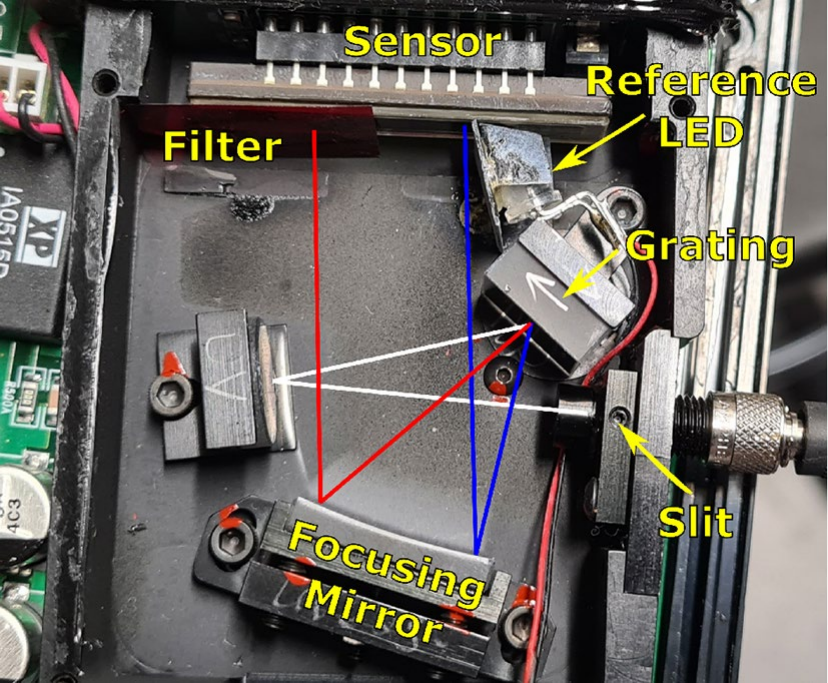
The modified spectrometer, probe light (white) incident from fiber-optic mounted to the 50 μm slit

The stock grating in the spectrometer (Fig. 5) was replaced with 600 lines/mm grating (Thorlabs GR25-0605) and an “order-sorting” 650 nm long-pass filter was installed at the appropriate position. A small LED was mounted inside the spectrometer next to a masked-out portion of the sensor, this LED was driven by a portion of the same current pulse used to drive the probe LED and served as a reference signal that normalises both current fluctuations in the probe flash circuits as well as variations in the pre-amplification circuit seen in the spectrometer. The spectrometer was spectrally calibrated using a neon calibration lamp (Newport, 6032).

### Xenon Flasher construction

The flash-lamp unit was designed for “compact” type xenon arc-discharge flash-lamps as per the Hamamatsu 10 W L464X series, or similar parts from other manufacturers. In this application, the flash-lamp power supply section is designed to simultaneously charge a 100 nF capacitor to 1000 V and a 220 nF capacitor to 135 V within a time frame to allow flash operation of up to 20 Hz repetition rates when used with these flash-lamps. The control input was originally opto-coupled and driven from a microcontroller output pin (5 V level). This enabled the controller to begin the charging cycle, wait for a period sufficient to attain a full charge level, and then turn the controller off whilst simultaneously triggering the flash-lamp discharge. This method meant that the charger was only running on a per-pulse basis as needed. The trigger section has been upgraded from the original on-board input-isolation opto-coupler to fibre-optic transmitter and receiver modules with a fibre-optic connecting cable which gives both improved timing stability and removes EMC conduction issues experienced with a coax trigger cable. The input supply voltage range is from 9 to 40 V DC. The current consumption varies from 60 to 200 mA when in operation. At higher voltages, a higher repetition rate may be used, although active cooling of the lamp enclosure may be required. The repetition rate for the instrument presented here was limited to 10 Hz.

**Fig. 6:**
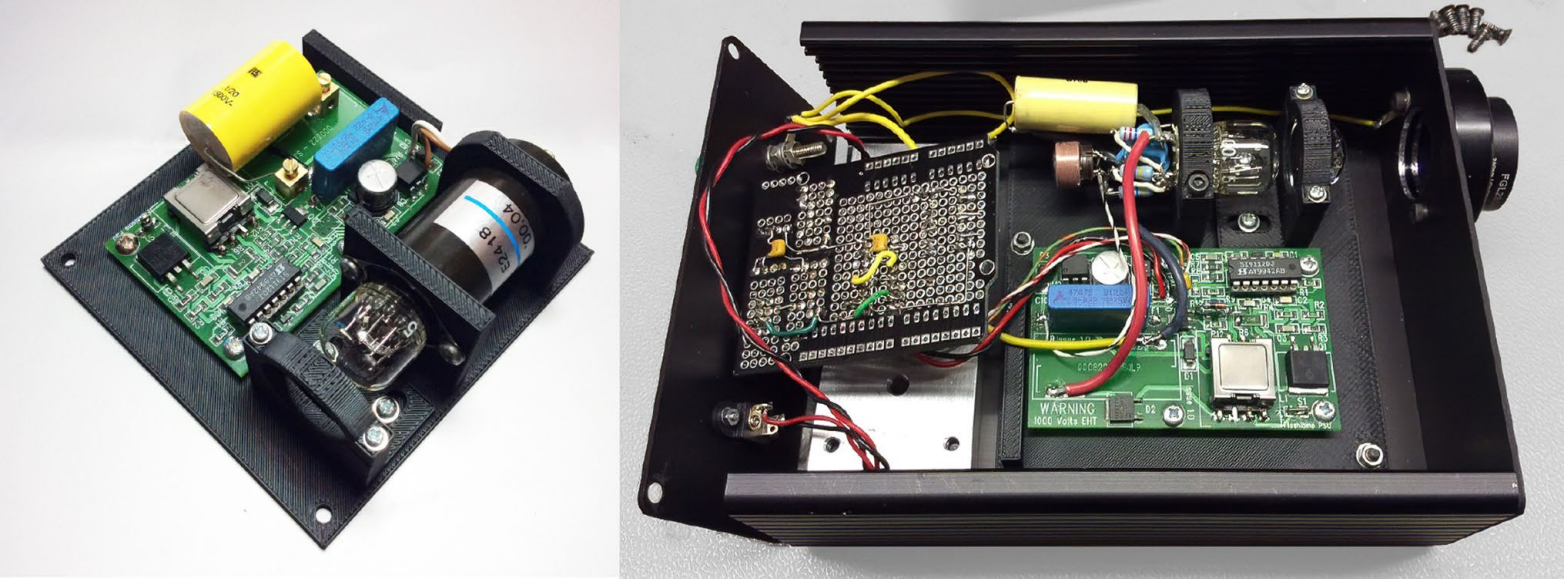
Xenon flash-lamp sub-assembly mounted on a 3D printed ABS plate using the commercial Hamamatsu trigger pack (left) and the fully assembled unit including fiber-optic trigger board and custom flash-lamp trigger pack mounted inside aluminum case (right)

The flash-unit is housed in an extruded aluminum case (Fig. 6) to provide shielding of the discharge EMI pulse, with a 3D printed ABS sub-plate which mounts the main circuit board, xenon flash-lamp, and collimating lens as a single assembly.

The xenon flash-lamp may use the Hamamatsu socket type E2418 or a custom socket may be constructed on a B9A vacuum tube base. The components used here must be rated for the full 6 kV trigger pulse voltage, not just the 1 kV charging voltage.

**Fig. 7:**
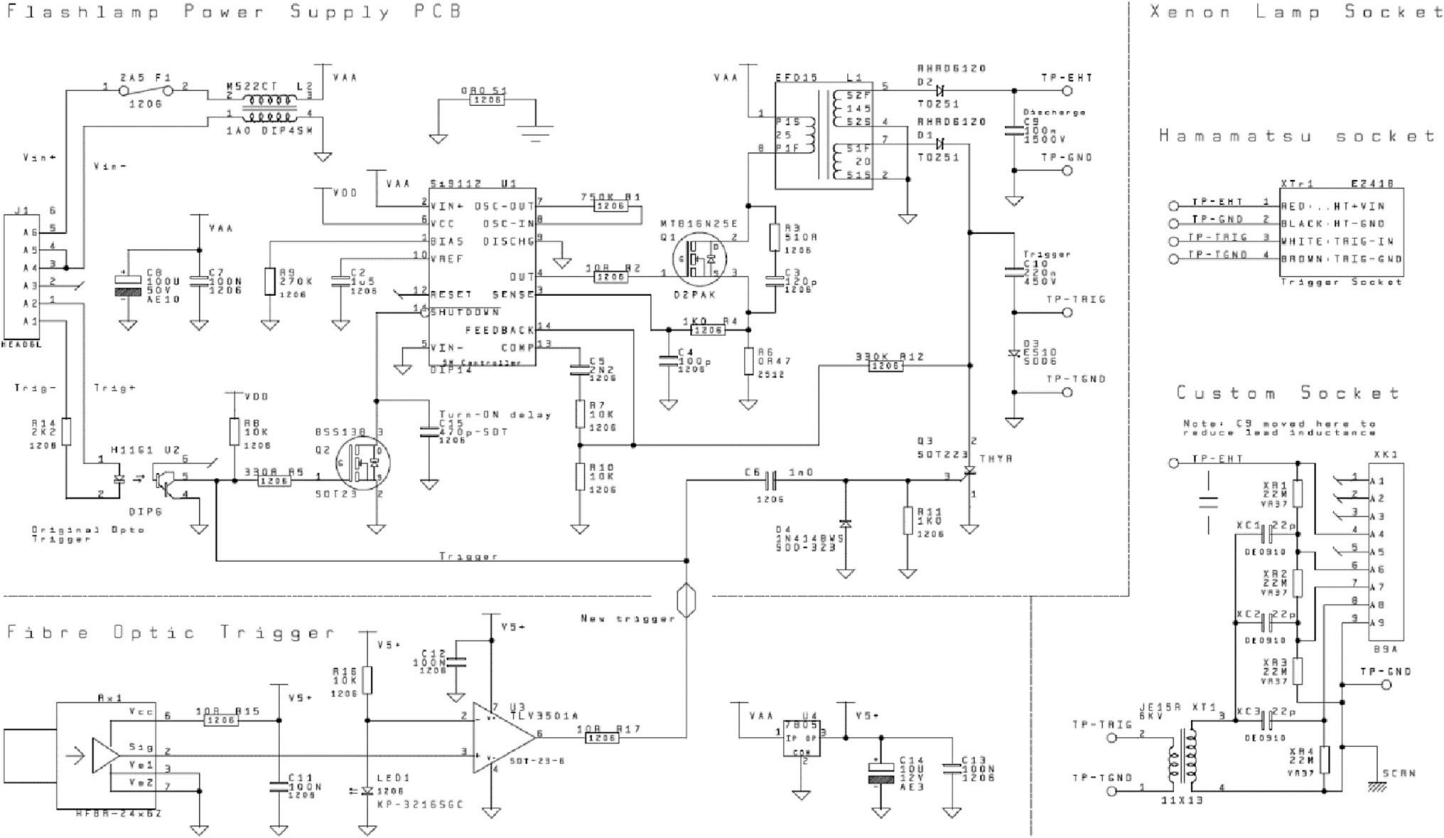
Circuit schematic of custom xenon flash-lamp unit. Full list of parts is shown in Table S1

The circuit diagram of the unit is depicted in Fig. 7. The PSU is designed around a Si9112 switch-mode controller IC (U1). It is noted that the Si9112 is now obsolete at the time of writing but can be substituted with a newer part such as the LT3757 controller, which has a modified pin numbering but equivalent function (please see SI for updated circuit schematic). The Si9112 operates at 20 kHz switching frequency (*F*_Osc_) set by a resistor (R1), with a current limiting resistor (R6) of 0.47 ohms. This is chosen to limit the charge pulses to that approximately half the core saturation time and current. (N.B. The PSU can operate at 100 Hz with a 24-V supply and with *R*6 reduced to 0.22 ohms).

The output switching NMOS switch (Q1) is specifically chosen to minimize the internal device resistance (RDS on) whilst providing protection in an out of range situation (e.g. if the feedback loop is not connected) and will minimize the maximum EHT voltage to approximately 1500 V, which is the transformer 6 × step-up ratio of the MOSFET avalanche voltage. In this situation, the device is dumping significant amounts of flyback energy and will become warm. Although the Si9112 can drive directly, the *F*_Osc_ frequency is low so turn on/off losses are not a problem. An EFD15 ferrite “kit” with a 3C90 core is used for the inductor (*L*_1_) flyback-transformer. This has three windings: a 25 turn primary that charges the inductor and two secondaries of 20 and 145 turns across which the output voltages are developed from the back EMF of the core magnetization collapse during flyback. A gap of 0.15 mm is used to prevent core saturation.

**Fig. 8:**
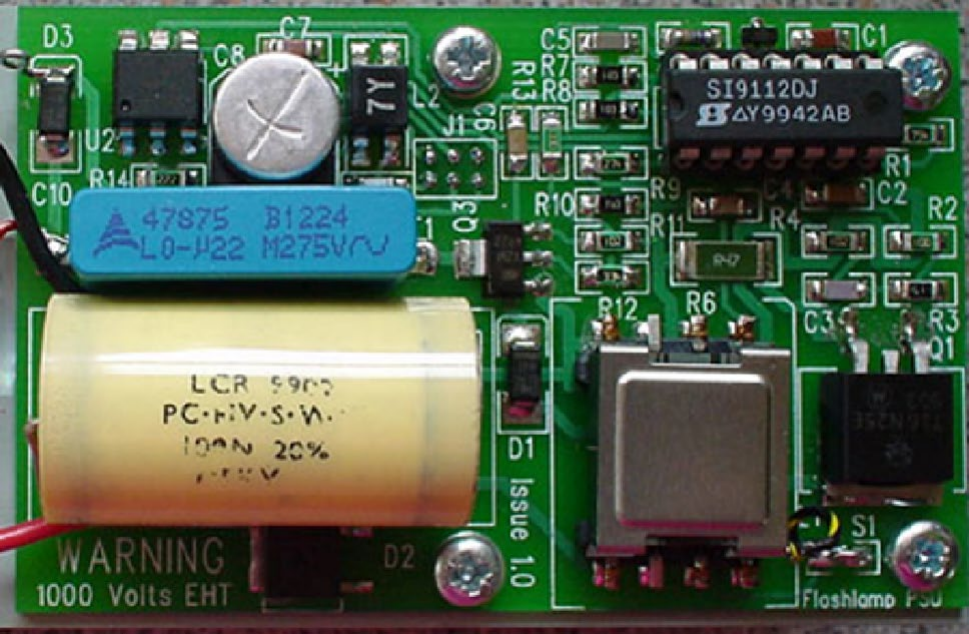
Xenon flasher main PCB

The flash-lamp is triggered by an input pulse to the fibre-optic receiver (Rx1) and high-speed comparator (U3) via a capacitor (C6) to the thyristor (Q3). The resistor (R11) forms a short time constant and the diode (D4) snubs the reverse edge of the trigger pulse. This pulse also turns on the MOSFET (Q2) which activated the controller (U1) /shutdown control input. A capacitor (C15) provides a turn-on delay of approximately one millisecond to ensure that the charge capacitor (C9) is sufficiently discharged post-flash to not hold the thyristor (Q3) in an ON state. The JE15R Trigger Coil (XT1) had its primary and secondary windings separated. This part may be substituted with part ZS1052-1(H), noting the pin numbers are different. The trigger is a negative pulse. We note that the current circuit is not isolated, and that zero volts is referenced to supply negative (see Fig. 8).

The main PCB circuit board is 79 × 50 mm in standard FR4 material. All components except J1 are surface mounted. The Si9112 switch-mode controller is available in a surface-mount package but for ease of development the initial design choice opted for a DIP socketed part to allow for easy replacement; the socket itself is surface mount.

The discharge capacitor (C9) (100 nF, 1500 V) and trigger capacitor (C10) (220 nF, 275 Vac) are normally through-hole mounted but have their leads formed for surface mounting. These capacitors should be secured to the PCB with silicone adhesive after final unit assembly and successful testing. We note that in the upgrade to the fibre-optic trigger and custom trigger-pack the discharge capacitor (C9) was moved to the flash-head to reduce lead inductance. The leads to the trigger pack are soldered directly onto these capacitor mounting pads, with leads being kept short. The HV trigger pack lead should be electrically insulated with PTFE or equivalent. Care should be used in the HV sections, especially if using solder paste, to ensure no electrical bridging paths are left under the components. A guide to testing the xenon flash-lamp can be found in the supplementary materials.

### Xenon flash-lamp as a pump source

**Fig. 9:**
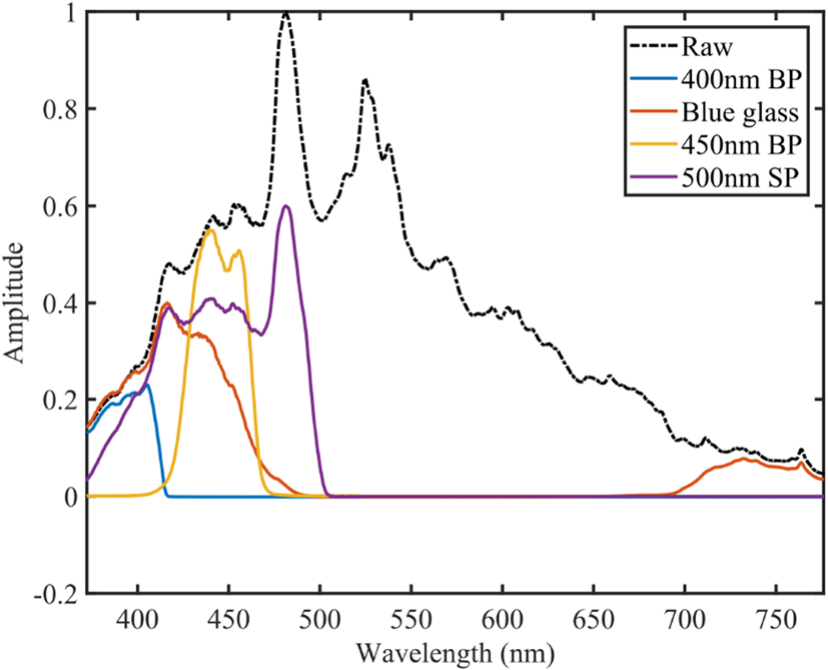
Spectral profile of xenon flash-lamp, using AR coated condenser lenses and various filters specifics of each filter is listed in Table 1

The emission from the xenon arc is broadband extending from 200 to 2000 nm, the visible part of the spectrum is shown in Fig. 9. Xenon arc light was collected and refocused using two *f* = 20.1 mm aspheric condenser lenses (Thorlabs ACL2520U-A), which gave a 1:1 imaging of the ~ 3 × 1 mm arc. The “A” antireflection coating limits the wavelength to 350–750 nm but minimizes losses in this region. The pulse energy measured at the sample plane in this arrangement is > 2 mJ, this allows the use optical filters to isolate a portion of the emission (Fig. 9) thereby creating pump pulses which spectral target specific photoreactions while at the same time maintaining sufficient pulse energy to drive mOD differences. Examples of some of the optical filters used and corresponding pump pulse energies are shown in Fig. 9 and Table 1. The focus was positioned at the entrance window of the sample cuvette to maximize the emission coupled into the sample as well as reduce pump scatter to the measurement spectrometer. Note: The range of pump wavelengths can be increased to 300–2000 nm using the uncoated variant of B270 glass condensers (Thorlabs ACL2520U) at the cost of 20% reduction in maximum pump power.

**Table 1:** Pulse energy values for xenon flash with the use of different filters to isolate areas of the blue part of the visible spectra

Filter	Range (nm)	Supplier	Part #	Pulse Energy (μJ)
400 nm BP	380–420	Thorlabs	FBH400-40	32.2 ± 0.1
Blue glass	315–445, 715–1095	Thorlabs	FGB25	255.8^a^ (100)
450 nm BP	430–465	Thorlabs	FB450-40	56.4 ± 0.1
500 nm SP	375–500	Thorlabs	FES0500	75 ± 0.2

^a^Blue glass filter also allows significant transmission in 700 nm + region, value in brackets is the estimated energy in the blue part of the spectra

### Triggering controls

The entire system was locked to the ~ 20 Hz repetition rate of the spectrometer, with the primary trigger taken directly from the spectrometer board. The probe source was operated at full the repetition rate while the xenon lamp and data collection oscilloscope were triggered at 10 Hz allowing for the collection of interleaved pump-off and pump-on spectra. The triggering rate was halved using a flip-flop circuit. Two analogue delay generators (Aim-TTi, TGP1110) were used to control the relative delays between the pump and probe pulses as well as the probe pulse width. Delays and optical pulse width were confirmed by measuring the scatter from the flasher and probe using a photodiode. The temporal jitter of the pump and probe with respect to the primary trigger was found to be 210 ns and 20 ns, respectively. Both pump and probe sources were powered by individual 6 V lead-acid batteries (RS PRO 537-5438) to isolate them from mains AC noise, reduce ground loops and other electrical noise from the pulsed sources being picked up by the measurement oscilloscope.

### Temporal performance

**Fig. 10:**
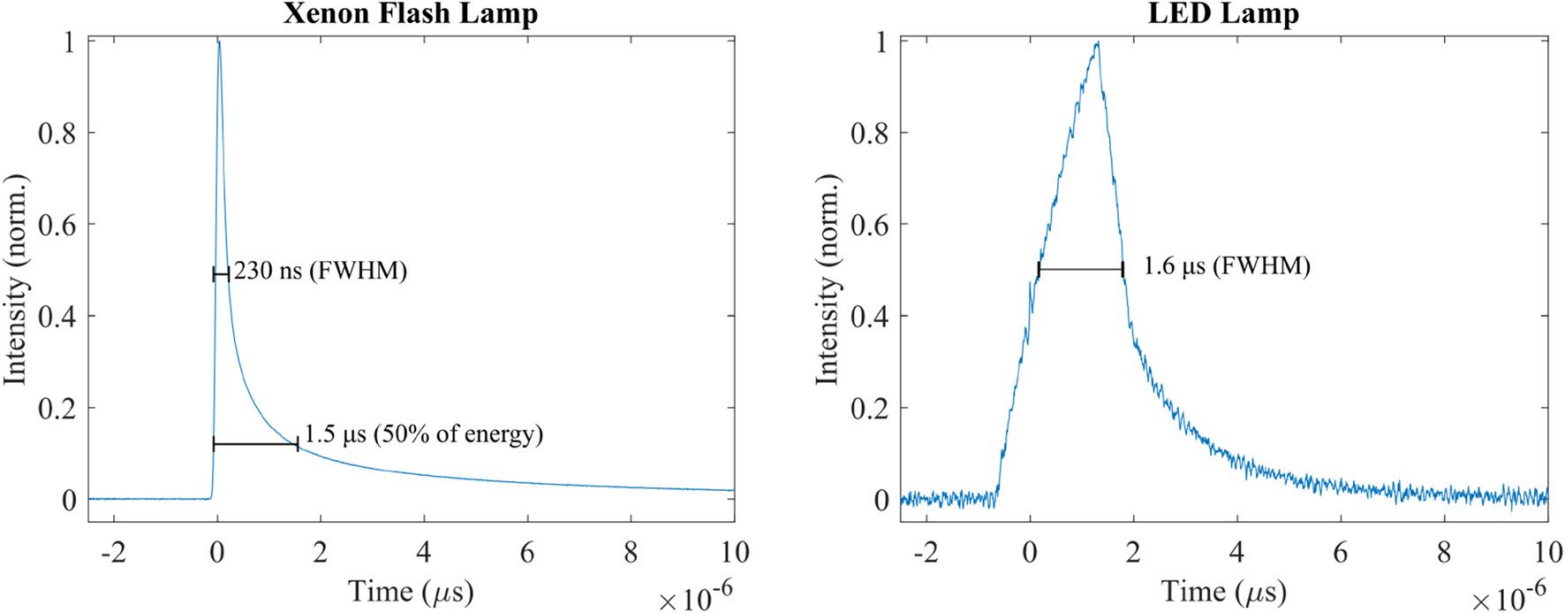
Temporal profile of the xenon flash-lamp pump (left) and typical white light probe (right) collected from scatter on a photodiode and oscilloscope. Note: xenon pulse shown was collected using 400–500 nm bandpass filter which reduces the pulse length owing to the later emissions being dominated by red/IR radiation

The temporal profiles of both the pump and the probe were measured by collecting scatter on a photodiode (Fig. 10). The xenon flash demonstrates the expected behavior with a sharp rise (~ 100 ns) and much longer complex exponential decay consistent with a combination of capacitor discharge and current dependent resistance of the xenon arc. The pulse width is measured at 230 ns (FWHM), however, due to the asymmetric pulse shape this is an underestimate. We take the width of the first 50% of the integrated pulse (for values > 0.5% of the peak value) as a more accurate 1.5 ± 0.5 μs width, this corresponds to when 50% of the pulse energy has been deposited into the sample. A future improvement for the xenon flasher could utilize a second MOSFET triggered after the peak of the pulse to dump the remaining charge, bypassing the lamp, which would remove the long tail and in principle produce ~ 200 ns pulse for this particular arc length (3 mm). The probe source width can be controlled by the width of the TTL trigger pulse used. Figure 10 shows a typical probe pulse length that was used for data collection chosen based on the width of the pump pulse and to provide sufficient illumination to fill the dynamic range of the spectrometer and, therefore, maximize the sensitivity of the instrument. This pulse length limited the fastest pump-probe delays to ~ 2 μs while the longest was ~ 50 ms limited by the instrument cycle time. Long pump-probe delay can be achieved by further reducing the repetition rate of the xenon flasher and selecting the correct probe spectra, however, this will increase the data collection time. Shorter sub-microsecond probe pulses could be achieved but require higher driving voltage to maintain the same brightness. However, if shorter pulses with lower brightness are used more TAS pump-probe cycles may be needed to accumulate sufficient sensitivity.

### Data collection, processing and performance

Oscilloscope traces were typically collected 2.5 mega samples per second and 200 ms/div, which corresponded to 19 full pump-on/pump-off spectra pairs per trace and ~ 44 samples per nm in the spectra allowing a degree of smoothing to be applied without the loss of spectral resolution. To discriminate between pump-on and pump-off spectra the oscilloscope was triggered using the ~ 10 Hz pump trigger. The “alarm” feature in the Picoscope software program was used to save spectra once the oscilloscope memory buffer was full. For each dataset, it was necessary to collect several negative time points to both separate transient absorption signals from pump-induced fluorescence and/or scatter and confirmed that the sample had not degraded or changed between data collections. Spectra were processed using MATLAB scripts to average the data, apply calibrations and referencing using the signal from the small LED inside the spectrometer. Due to the large data size and computer RAM limitations, it was necessary to employ a rolling average of spectra using the following weighted-average formula:

2 $$\overline{S}_{T} = \frac{{n_{1} \overline{S}_{1} + n_{2} \overline{S}_{2} }}{{n_{1} + n_{2} }},$$

where $\overline{S}_{T}$ is the total averaged spectra constructed from two multiple averages $\overline{S}_{1}$ & $\overline{S}_{2}$, each made from *n*_1_ & *n*_2_ measurements, respectively. Pump-on and pump-off spectra were averaged separately and then transient absorption differences were calculated using:

3 $${\Delta }A = - \log_{10} \left( {\frac{{S_{{{\text{ON}}}} }}{{S_{{{\text{OFF}}}} }}} \right).$$

Due to a slight mismatch between the timing of the oscilloscope and spectrometer clocks it was necessary to use simple interpolation to shift one of the spectrum by 0.9 sample points to ensure proper overlap of the two spectra. Finally, the negative pump-probe delay datasets were compared and if in agreement averaged together and subtracted from the others to give the transient absorption spectra.

**Fig. 11:**
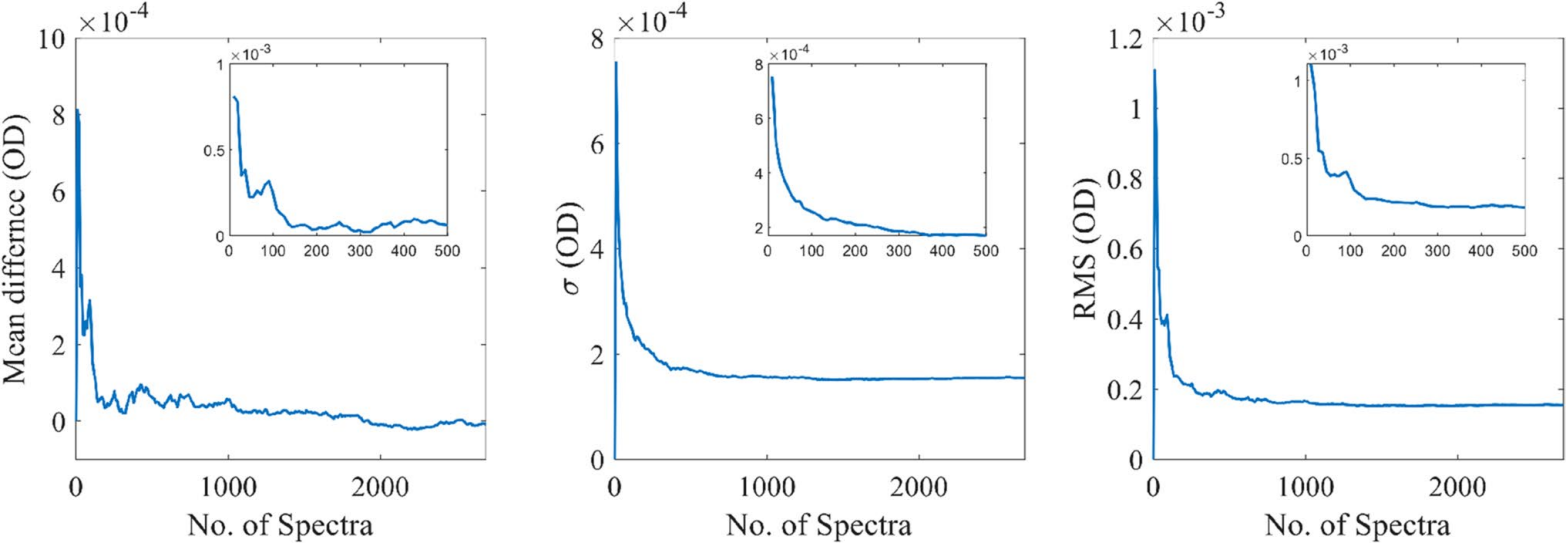
Statistical performance of the transient absorption spectrometer with number of spectra collected. Showing the mean un-pumped difference (left), standard deviation (middle) and root mean squared (right), each calculated for the FWHM range of the probe spectra. Showing that the statistical noise of the instrument plateaus after ~ 500 pump-on/pump-off spectral pairs corresponding to 1000 measurements or 100 s of data collection

Data collections on a water-filled cuvette showed that the empty spectra noise plateaued within ~ 500 spectra (or 1000 measurements) giving a sensitivity of ~ 0.15 mOD, the current noise limit of the instrument (Fig. 11), while a sensitivity of ~ 0.25 mOD was already reached within 100 measurements.

**Fig. 12:**
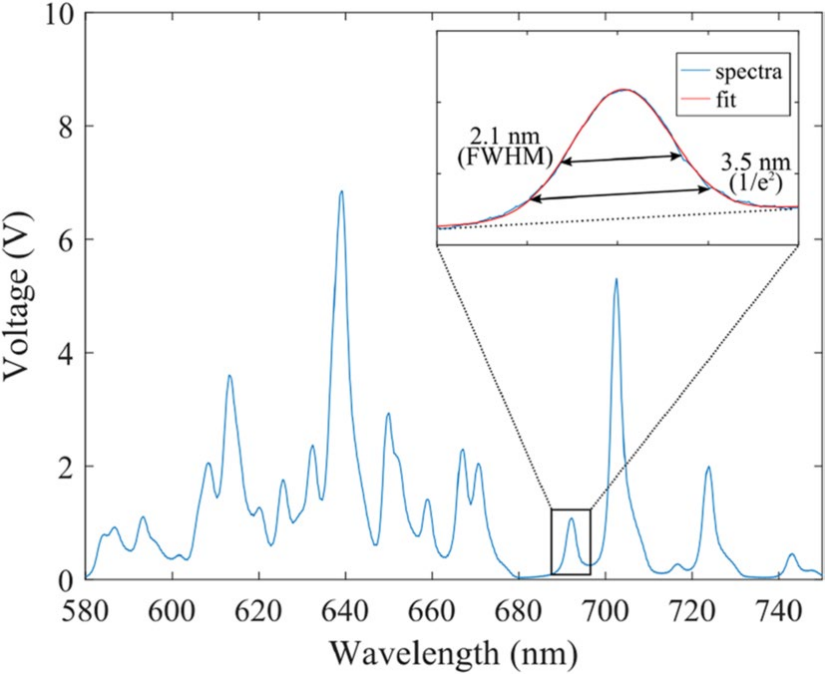
Neon calibration lamp spectra measured using the instrument spectrometer. Insert: zoom in on the 693 nm emission line (blue) and a Gaussian fit (red), which recovered a spectral line width and hence the spectral resolution of 3.5 nm (1/*e*^2^). Note: a linear component was added to the fit to correct for the baseline slope (dashed black) cause by the nearby stronger 703 nm line

As the data were oversampled by the oscilloscope it was possible to perform a degree of smoothing to the data while staying below the single-pixel limit. Figure 12 shows the spectra collected from a calibration neon lamp, a Gaussian fitting (inset) of the relatively isolated Ne I 693 nm line indicates the spectral resolution of the instrument is ~ 3.5 nm (1/*e*^2^) corresponding to ~ 150 sample points which sets the formal limit for smoothing the oversampled spectra. Data smoothing is discussed further in the supplementary material.

## Results and discussions

### Congo red experiment

**Fig. 13:**
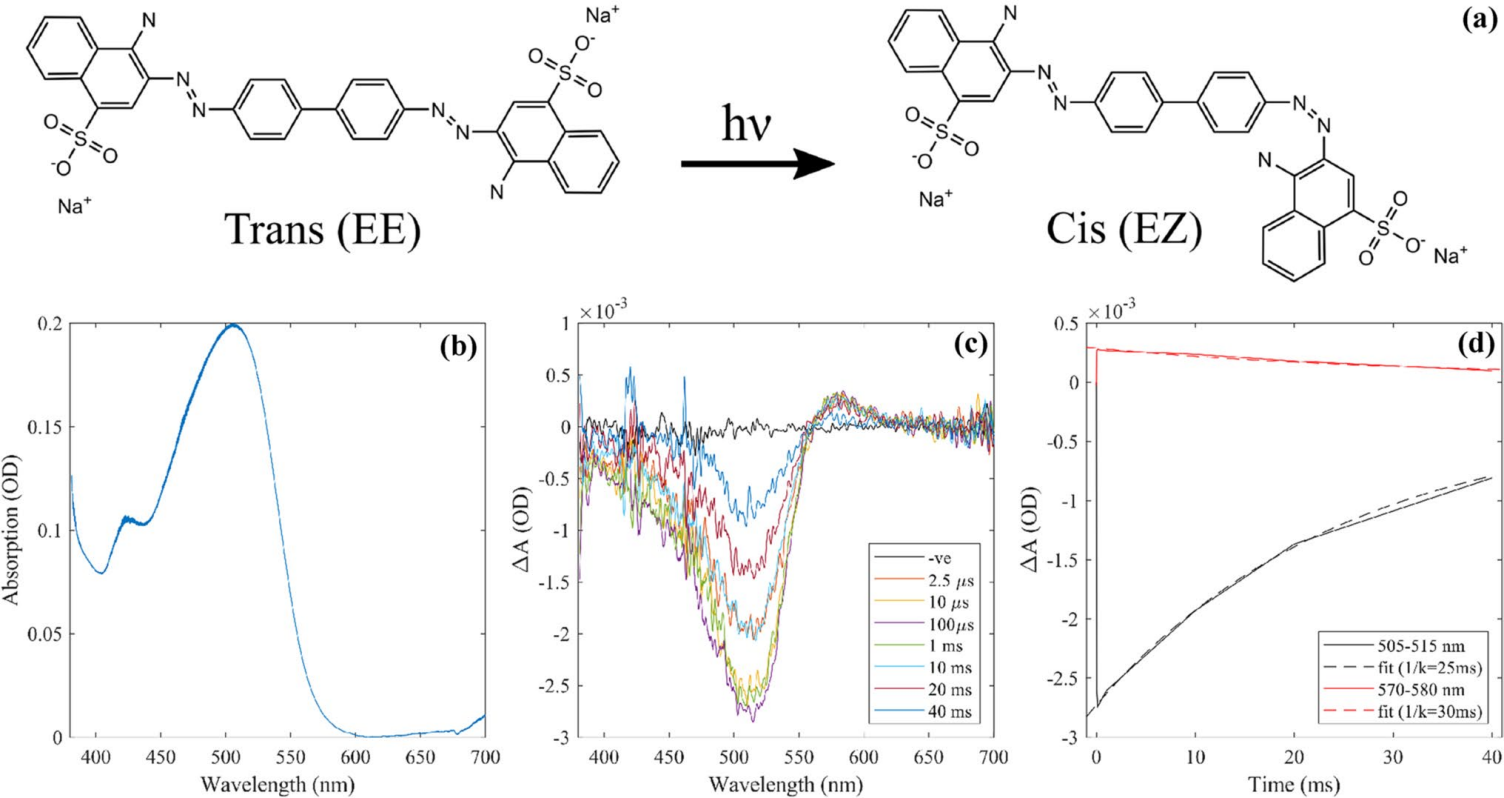
**a** Congo red photoisomerisation reaction. **b** Absolute time-resolved absorption spectra calculated **c** time-resolved difference absorption spectra collected at various microsecond and millisecond delays. A smoothing filter window corresponding to 3.5 nm was used. A GSB is seen at 400–550 nm and an induced absorption at 550–600 nm both of which peak at ~ 100 μs then decay on the 10’s of millisecond time scale. **d** Exponential fitting of the temporal lineouts of these signals recover decay constants ~ 30 ms

Congo red is the sodium salt of benzidinediazo-bis-1-naphthylamine-4-sulfonic acid, a diazo dye. Upon excitation with 500 nm light it undergoes trans → cis photo-isomerisation to a thermally unstable cis confirmation (Fig. 13a). The rate at which Congo red and other similar Azobenzene derivatives thermally recover has been shown to depend highly on the polarity of the solvent used [25], this reaction also can catalyzed by the presence of hydroxide ions [26]. A 50 μM (0.003% *w*/*v*) Congo red (Sigma) solution was prepared in 80% ethanol in water, 0.2 mM NaOH, 2.5 mM Tris pH 7. The three windowed micro-volume cuvette was filled with 200 μL of solution and sealed to avoid evaporation. The 3 mm path length of the cuvette gave a peak absorption of 0.2 OD at 500 nm (Fig. 13b). The sample was flashed with the unfiltered xenon lamp to drive photoisomerisation at a variety of pump-probe delays. The transient absorption spectra are shown in Fig. 13c. A ground state bleach (GSB) of -2.7 mOD can be seen between 400 and 550 nm and an induced absorption 0.3 mOD at 550–600 nm. Both features peak at around 100 μs and decay on the 10’s of ms timescale. Fitting of simple exponentials to the slower time points of these spectral features (Fig. 13d) recover decay constants of 25–30 ms. Further accuracy could be achieved by simply increasing the number time steps over this range. This measurement demonstrates the ability of the instrument to measure 0.15 mOD signals (induced absorption). The shape and form of the TA signals for Congo red are consistent with reported in femtosecond transient absorption spectroscopy [25], while the timescales are far slower due to the polar solvent.

An estimation of the Congo red photoswitching yield can be made from the TA data. Given the peak absorption at 500 nm of 0.2 OD and maximum GSB at the same wavelength corresponds to an excitation of 1.4% of molecules in the sample. The molar attenuation coefficient of Congo red is reported to be 62,600 M^–1^ cm^−1^ at 500 nm in solution [27], taking the peak absorption of 0.2 OD and cuvette volume of 3 × 3 × 3 mm (1.07 × 10^–5^ L) corresponds to ~ 1.7 × 10^14^ molecules present in the sample. The sample was excited using the unfiltered white light source, from a comparison of the Congo red absorption spectra with the xenon lamp spectral profile (as seen in Fig. 9) we estimate a 60% useful pump photons. Using the measured absorption spectra and pulse energy from the 450 nm bandpass filter we estimate ~ 500 μJ of pulse total pulse energy in the visible region and, therefore, 300 μJ of useful pump emission, corresponding to 7.22 × 10^14^ photons at the average absorption wavelength of 478 nm. By scaling the molar attenuation coefficient to the absorption and spectra overlap we recover an average effective molar attenuation coefficient of ~ 30,000 M^–1^ cm^−1^. Scaling this value to the average overlap between the pump spectra and absorption spectra gives an average value of ~ 29,000 M^–1^ cm^−1^ and corresponding to an average absorption of 0.09 OD. We can, therefore, estimate that a total ~ 1.34 × 10^14^ photons were absorbed and ~ 78% of molecules were excited. Therefore, the estimated quantum yield for this reaction under our conditions is ~ 2%, which demonstrates the device’s ability to measure low quantum yield dynamics.

### Commercial system comparison

For comparison, a commercial instrument is the LP980 Spectrometer from Edinburgh instruments, the “Kinetic” model of the system uses a monochromator and photomultiplier tube (PMT) target and measure transients at a specific wavelength. Repeated measurements across the different wavelengths to build up the pump-probe spectra. While the “Spectral” model upgrade uses a gated ICCD detector and allows measurements from 3 ns  with a spectral range of 230–930 nm is the best comparison with our instrument. The instrument uses a very bright and potentially actinic xenon lamp as the probe source and requires a high-power nanosecond laser system to serve as the pump. The sensitivity for time-gated spectral measurements of the instrument is similar at the < 1 mOD level [28]. Besides the substantial difference in costs and small footprint 0.5 m × 0.5 m, our instrument has a different design which does not require the pump laser, the white light probe is not actinic and the excitation source ensures linear optical response.

### Future improvements

The temporal resolution of the instrument is currently limited by a number of factors that can be improved potentially to sub-microsecond measurements. As previously stated, the long emission tail of the xenon flash could be improved through the use of a second MOSFET timed to dump the remaining charge stored in the flashers capacitors after the peak of the pulse and produce a more Gaussian/Top hat shape. The timing jitter between the electronic trigger and the xenon lamp “firing” we believe is partially caused by the recharging time of the capacitors not being full synchronized to the cycle time of the instrument. The capacitors are fully charged well within the 50 ms interval time, resulting in them “idling” at full charge where the passive discharge will occur, to maintain the peak charge the charging circuit repeatedly tops up the charge and the total charge inside the capacitors oscillates around the target voltage. If this cycling of charge is not synchronized to the repetition rate of the instrument then the charge that is stored at the moment of discharge will vary slightly which may result in instability in pulse energy and temporal jitter. This could be corrected through the use an Arduino or other microcontroller to synchronize the charge cycle to the instrument repetition rate.

The system currently uses a L464X series xenon flash lamp, Hamamatsu also produces the L463X which includes a reflector built into the lamp assembly that would greatly increase the amount of light captured from the arc discharge, effectively increasing the pump pulse whilst not impacting pulse length. The converging/focusing variant of this reflector lamp would further improve the setup by removing the need for an external imaging system and its associated losses. The synthesis of all the timing and trigger pulses could alternatively be done with a microcontroller such as an Arduino or Teensy using an interrupt function for the trigger, but the analog systems used here are also convenient and inexpensive. The LED high current flasher could be driven at increased voltage (10–20 V) to generate shorter flash durations down to approximately 0.5–1.0 microseconds although care should be taken to avoid overloading and destroying the LEDs. A further reduction in measurement noise could be achieved by actively cooling of the CCD sensor which is well known to dramatically reduce the dark current. The spectrometer used here utilizes the inexpensive Sony ILX511 sensor which has sufficient performance for the demonstrated application of transient absorption measurements (Fig. 11). The high sensitivity of the sensor however allows the detection of the microsecond pulsed LED light driven under high current pulses from the capacitor bank. The resulting photometric accuracy is shown to be sufficient. The performance of more expensive spectrometers would further improve the performance of the system, using for example the TEC-cooled Hamamatsu S7034-1007S, which is a 1024 × 122 pixel back-thinned FFT-CCD sensor that can be used in binning mode with the illumination of a large area. A commercial pre-amplifier and data acquisition electronics C7044 and C7557-01 (Hamamatsu), combined with a suitable spectrometer achieves increased performance from the higher full well depth capacity, SNR, sensitivity and lower noise, yet should still be able to exploit the LED flash probe intensity presented here.

## Conclusion

We have demonstrated an open source, open hardware TAS setup that is capable of measuring 0.15 mOD signals in the visible spectral region. The highly customizable and adaptable design of the setup allow it to be used to study a wide range of samples with dynamics on the microsecond to millisecond and also second timescale. The instrument design includes the ability to measure and analyze transient absorption spectra with good sensitivity even for low quantum yield processes. The microsecond time resolution supports the measurement of the linear optical response and is applicable to a wide range of experimental problems, including photoisomerization, triplet state dynamics, electron and proton transfer reactions and thermally driven relaxation processes in chemistry and biochemistry research.

## Supplementary Information

Below is the link to the electronic supplementary material.

Supplementary file1 (DOCX 1512 kb)

## Data Availability

Datasets available.

## Abbreviations

ABS: Acrylonitrile butadiene styrene
ADC: Analogue to digital converter
CCD: Charge-coupled device
CW: Continuous wave
DIP: Dual in-line
EMI: ElectroMagnetic interference
ETH: Extra high tension
GSB: Ground state bleach
HV: High voltage
LED: Light-emitting diode
MOSFET: Metal oxide semiconductor field effect transistor
NMOS: N-type MOSFET
OD: Optical density
RDS on: Drain-source on resistance
PSU: Power supply unit
TAS: Transient absorption spectroscopy
TA: Transient absorption
TTL: Transistor-transistor logic
