# Supplementary material for: Open hardware microsecond dispersive transient absorption spectrometer for linear optical response
Source: Photochem Photobiol Sci. 2021 Nov 8;21(1):23–35. doi: 10.1007/s43630-021-00127-6 (PMC8799588; doi:10.1007/s43630-021-00127-6)
Supplement: Supplementary file 1 — Supplementary file1 (DOCX 1512 kb) [file 43630_2021_127_MOESM1_ESM.docx]

Open hardware microsecond dispersive transient absorption spectrometer for linear optical response

Christopher D.M. Hutchison^1^, Susan Parker^2^, Volha Chukhutsina^1^ and Jasper J. van Thor^1^*

^1^. Department of Life Sciences, Imperial College London, London SW7 2AZ, United Kingdom,
^2^. QOLS Physics Group, Blackett Laboratory, Imperial College London, London SW7 2BW, United Kingdom

*Corresponding author e-mail: [j.vanthor@imperial.ac.uk](mailto:j.vanthor@imperial.ac.uk)

# Supplementary materials


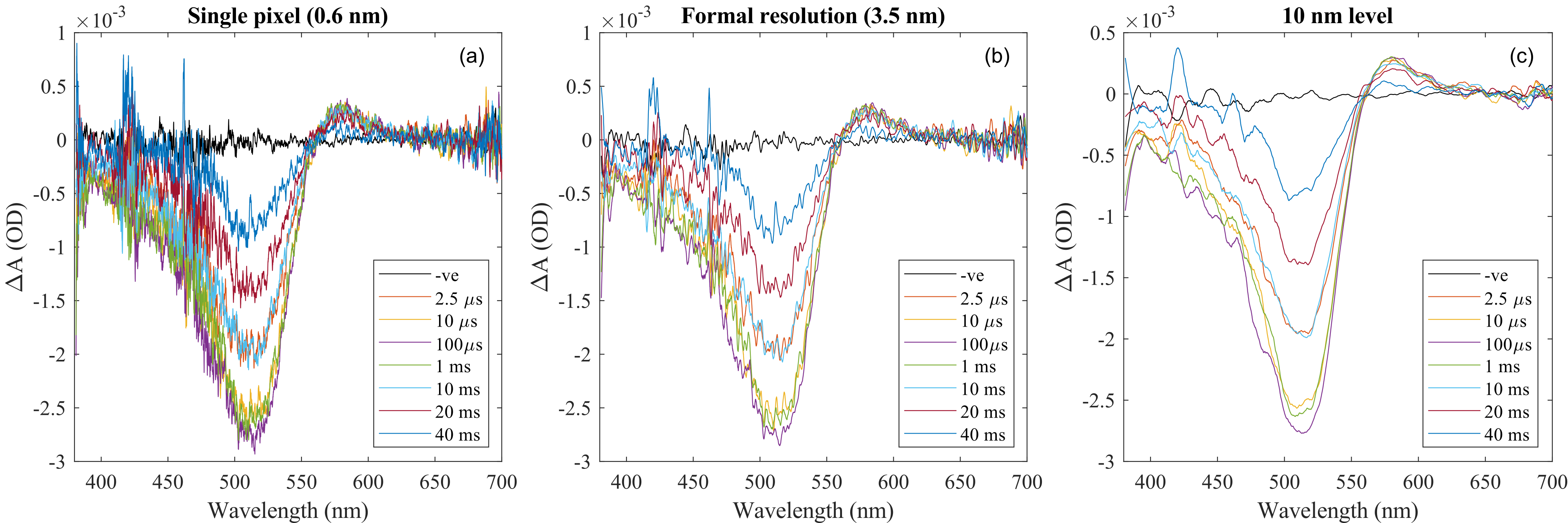


Figure S1. Congo red transient absorption spectra collected on the TAS instrument shown at different sizes of smoothing widow width, corresponding to (a) single pixel, (b) the formal spectral resolution calculated from the neon calibration lamp (Figure 12 main text) and (c) the 10 nm level corresponding to minimum estimated spectral width of feature corresponding to a room temperature measurement.

Table S1. Xenon flash-lamp unit circuit parts list

| Part No: | Package | Description | Value | Volt/Amp |
| --- | --- | --- | --- | --- |
| R1 | 1206 | Resistor | 750K |  |
| R2 | 1206 | Resistor | 10R |  |
| R3 | 1206 | Resistor | 510R | 200V |
| R4 | 1206 | Resistor | 1K0 |  |
| R5 | 1206 | Resistor | 330R |  |
| R6 | 2512 | Current Sense Resistor | 0R47 | 1W |
| R7 | 1206 | Resistor | 10K |  |
| R8 | 1206 | Resistor | 10K |  |
| R9 | 1206 | Resistor | 270K |  |
| R10 | 1206 | Resistor | 10K |  |
| R11 | 1206 | Resistor | 1K0 |  |
| R12 | 1206 | Resistor | 330K | 200V |
| R13 | 1206 | Resistor | 0R0 |  |
| *R14* | *1206* | *Resistor* | *2K2* |  |
| R15 | 1206 | Resistor | 10R |  |
| R16 | 1206 | Resistor | 10K |  |
| R17 | 1206 | Resistor | 10R |  |
| C1 | 1206 | Ceramic | 100n | 16V |
| C2 | 1206 | Ceramic | 1u0 | 16V |
| C3 | 1206 | Ceramic | 120p | 250V |
| C4 | 1206 | Ceramic | 100p | 16V |
| C5 | 1206 | Ceramic | 2n2 | 16V |
| C6 | 1206 | Ceramic | 330n | 16V |
| C7 | 1206 | Ceramic | 100n | 50V |
| C8 | AE10.6 | Al El cap | 100uF | 50V |
| C9 | Axial Cylinder | HV Discharge Cap | 100n | 1500V |
| C10 | Radial Box | Trigger Cap | 220n | 450V |
| C11 | 1206 | Ceramic | 100n |  |
| C12 | 1206 | Ceramic | 100n |  |
| C13 | 1206 | Ceramic | 100n |  |
| C14 | AE3 | Al El cap | 10u | 12V |
| C15 | 1206 | Ceramic | 470p |  |
| U1 | DIP14 | PWM Controller | Si9112DJ |  |
| *U2* | *DIP6 - SM* | *Opto Isolator* | *H11G1* |  |
| U3 | SOT-23-6 | High-Speed Comparator | TLV3501A |  |
| U4 | SOT-233 | Voltage Regulator | 7805 | 5V |
| Q1 | D2-Pak | NMOS Power | MTB16N25E | 250V |
| Q2 | SOT23 | NMOS Signal | ZVN3306F |  |
| Q3 | SOT223 | Thyristor | X0202MN | 600V |
| D1 | SMA | HV Diode | ES1D | 200V |
| D2 | TO251 | EHT Diode | RHRD6120 | 1200V |
| D3 | SMA | HV Diode | ES1D | 200V |
| D5 | SOD-323 | Diode | 1N4048 |  |
| F1 | 1206 | Fuse |  | 2500mA |
| S1 | 1206 | Not Fitted |  |  |
| J1 | Header | 6 pin 2 mm double row |  |  |
| Rx1 | HFBR | Fibre-Optic Receiver | HFBR-24x6Z |  |
| L1 | EFD15 | Bobbin |  |  |
| " | EFD15 | 3C90 Core pair |  |  |
| " | EFD15 | Clamp |  |  |
| " |  | 0.15 mm core spacer | Copper Foil |  |
| L2 | DIP4 - SM | Chip Inductor | M-522CT | 1A |
|  | DIP14 | SM turned pin IC socket | For U1 |  |
|  |  | PCB |  |  |
| XTr1 | Socket | Hamamatsu | E2418 |  |
|  |  | *or* |  |  |
| XK1 | Socket | Ceramic or PTFE Valve Base | B9A |  |
| XR1 | VR37 | High Voltage Resistor | 22M |  |
| XR2 | VR37 | High Voltage Resistor | 22M |  |
| XR3 | VR37 | High Voltage Resistor | 22M |  |
| XR4 | VR37 | High Voltage Resistor | 22M |  |
| XC1 | DE0910 | High Voltage Capacitor | 22p |  |
| XC2 | DE0910 | High Voltage Capacitor | 22p |  |
| XC3 | DE0910 | High Voltage Capacitor | 22p |  |
| XT1 | JE15R | Trigger Coil (Digikey) | ZS1052-1(H) | 6KV |


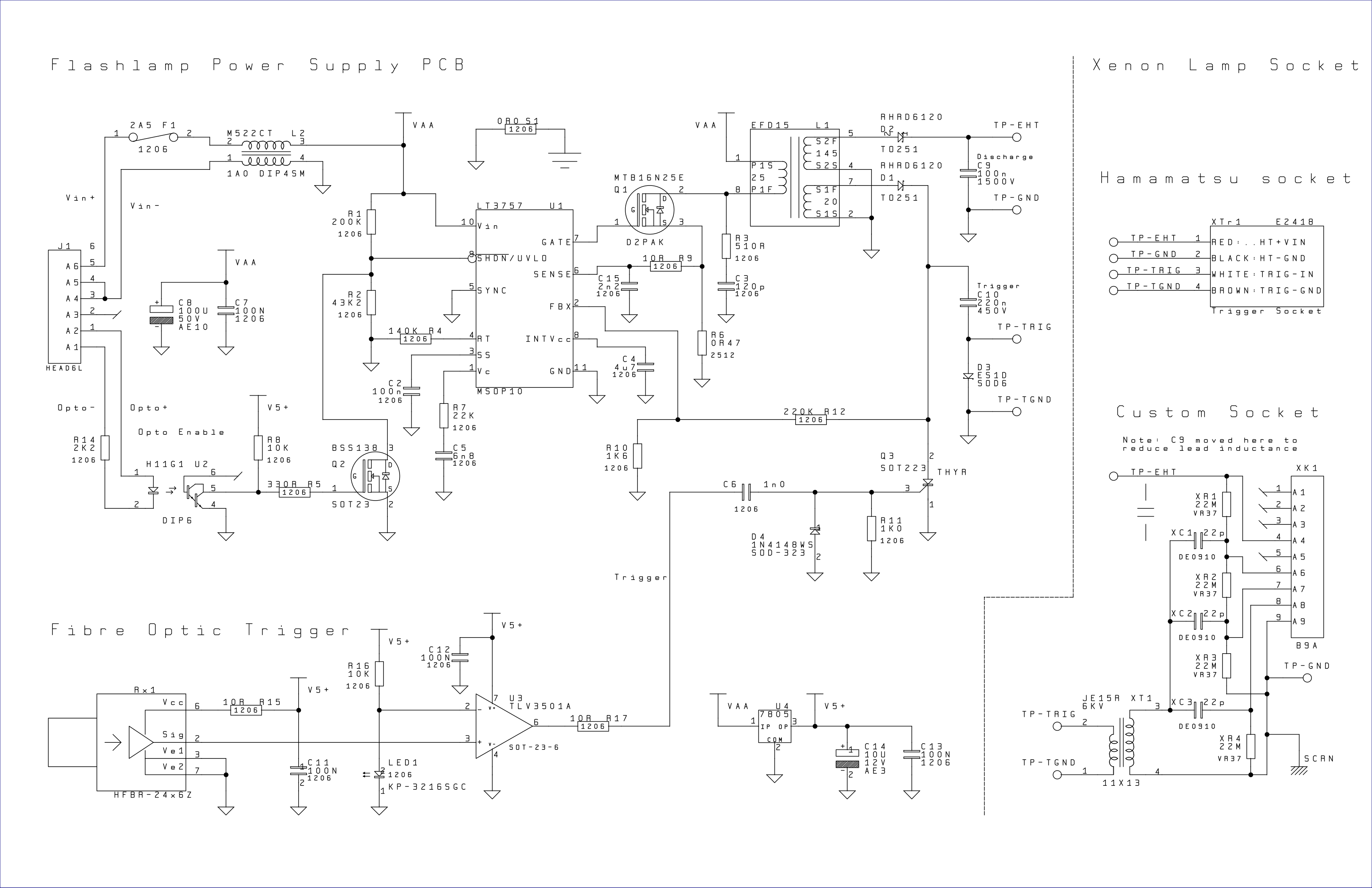


Figure S2. Alternative circuit diagram for xenon flasher using the substituted LT3757 controller. Modified parts list is shown in Table S2.

Table S2. Xenon flash-lamp unit circuit parts list for alternative LT3757 construction.

| **Part No:** | **Package** | **Description** | **Value** | **Volt/Amp** |
| --- | --- | --- | --- | --- |
| R1 | 1206 | Resistor | 200K |  |
| R2 | 1206 | Resistor | 43K2 |  |
| R3 | 1206 | Resistor | 510R | 200V |
| R4 | 1206 | Resistor | 140K |  |
| R5 | 1206 | Resistor | 330R |  |
| R6 | 2512 | Current Sense Resistor | 0R47 | 1W |
| R7 | 1206 | Resistor | 22K |  |
| R8 | 1206 | Resistor | 10K |  |
| R9 | 1206 | Resistor | 10R |  |
| R10 | 1206 | Resistor | 1K6 |  |
| R11 | 1206 | Resistor | 1K0 |  |
| R12 | 1206 | Resistor | 220K | 200V |
| *R14* | *1206* | *Resistor* | *2K2* |  |
| R15 | 1206 | Resistor | 10R |  |
| R16 | 1206 | Resistor | 10K |  |
| R17 | 1206 | Resistor | 10R |  |
| C2 | 1206 | Ceramic | 100n | 16V |
| C3 | 1206 | Ceramic | 120p | 250V |
| C4 | 1206 | Ceramic | 4u7 | 16V |
| C5 | 1206 | Ceramic | 6n8 | 16V |
| C6 | 1206 | Ceramic | 1n0 | 16V |
| C7 | 1206 | Ceramic | 100n | 50V |
| C8 | AE10.6 | Al El cap | 100uF | 50V |
| C9 | Axial Cylinder | HV Discharge Cap | 100n | 1500V |
| C10 | Radial Box | Trigger Cap | 220n | 450V |
| C11 | 1206 | Ceramic | 100n |  |
| C12 | 1206 | Ceramic | 100n |  |
| C13 | 1206 | Ceramic | 100n |  |
| C14 | AE3 | Al El cap | 10u | 12V |
| C15 | 1206 | Ceramic | 2n2 |  |
| U1 | DIP14 | PWM Controller | LT3757 |  |
| *U2* | *DIP6 - SM* | *Opto Isolator* | *H11G1* |  |
| U3 | SOT-23-6 | High-Speed Comparator | TLV3501A |  |
| U4 | SOT-233 | Voltage Regulator | 7805 | 5V |
| Q1 | D2-Pak | NMOS Power | MTB16N25E | 250V |
| Q2 | SOT23 | NMOS Signal | ZVN3306F |  |
| Q3 | SOT223 | Thyristor | X0202MN | 600V |
| D1 | SMA | HV Diode | ES1D | 200V |
| D2 | TO251 | EHT Diode | RHRD6120 | 1200V |
| D3 | SMA | HV Diode | ES1D | 200V |
| D5 | SOD-323 | Diode | 1N4048 |  |
| F1 | 1206 | Fuse |  | 2500mA |
| S1 | 1206 | Not Fitted |  |  |
| J1 | Header | 6 pin 2 mm double row |  |  |
| Rx1 | HFBR | Fibre-Optic Receiver | HFBR-24x6Z |  |
| L1 | EFD15 | Bobbin |  |  |
| " | EFD15 | 3C90 Core pair |  |  |
| " | EFD15 | Clamp |  |  |
| " |  | 0.15 mm core spacer | Copper Foil |  |
| L2 | DIP4 - SM | Chip Inductor | M-522CT | 1A |
|  | DIP14 | SM turned pin IC socket | For U1 |  |
|  |  | PCB |  |  |
|  |  |  |  |  |
|  |  |  |  |  |
| XTr1 | Socket | Hamamatsu | E2418 |  |
|  |  | *or* |  |  |
| XK1 | Socket | Ceramic or PTFE Valve Base | B9A |  |
| XR1 | VR37 | High Voltage Resistor | 22M |  |
| XR2 | VR37 | High Voltage Resistor | 22M |  |
| XR3 | VR37 | High Voltage Resistor | 22M |  |
| XR4 | VR37 | High Voltage Resistor | 22M |  |
| XC1 | DE0910 | High Voltage Capacitor | 22p |  |
| XC2 | DE0910 | High Voltage Capacitor | 22p |  |
| XC3 | DE0910 | High Voltage Capacitor | 22p |  |
| XT1 | JE15R | Trigger Coil (Digikey) | ZS1052-1(H) | 6KV |

**TESTING XENON FLASH LAMP:**

WARNING!

This circuit generates potentially lethal voltages. Use 4KV or higher rated probes.

1. Care must be exercised when operating the xenon flasher unit.
2. A special HV test area with dedicated equipment is recommended.
3. Always ensure that the feedback loop components are in place.
4. Start testing with a low rather than a high supply voltage. E.g. 12 volts.
5. Always ensure test lead grounds are connected before powering the circuit.
6. Use the same hand to hold a probe and change settings. Keep the other hand well clear of the circuit and any earth paths.
7. Do NOT use a static discharge mat or other items which may form a conductive ground path (even at a relatively high impedance) when powering the circuit.
8. Due large amount of hazardous UV light shield the flash-lamp and/or use protective glasses. Do NOT look directly at the arc with unprotected eyes.

Test Setup required Items:

1. 100+ MHz oscilloscope with 2 off HV (4KV) 100x probes.
2. Variable Power Supply, 9 to 40 volts, with metered V/I readout.
3. Current Meter, 0 to 500 mA, if not fitted to above.
4. Pulse Generator with TTL output and variable duty ratio.
5. HFBR-14x4 fiber-optic IR transmitter box, and matching fiber-optic cable.

Construct with Tx to match fiber cable termination in use, BNC connector and 50R series resistor. Being cautious add a reverse protection Schottky diode across the Tx emitter in case signal source has a negative component.

Testing Procedure

1. Turn on power, bring up to 12 volts and check current – there will be a current blip as the controller starts operation.
2. Check EHT and HV voltages. Adjust the value of R12 if necessary.
3. Set pulse generator to 20 Hz with a 1 uS ON time and 10V pulse into Fibre Tx box; flash lamp should flash! Check charge times for EHT and HV supplies.
4. With circuit running check Q1 gate drive for cycle period. The Si9112 should be running at 20KHz, but some variation may be seen from device to device. Adjust the value of R1 if needed.

Notes:

1. Charge times are longer for the Hamamatsu trigger packs to reach the final voltage after a flash-discharge than with the “custom” version.
2. The default condition for the PSU is active. I.e. it WILL operate immediately when power is applied.
3. The unit will operate at higher than specified frequencies, however the final EHT voltage may not be achieved. Over-running the control frequency will at some point cause "miss-fires" which is clearly audible.

The typical charging behavior expected is shown in Figure S1


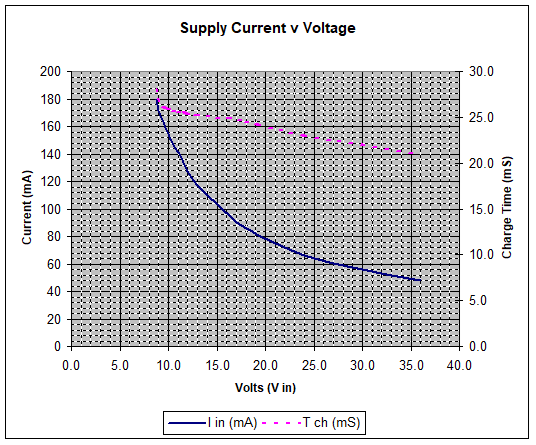


Figure S3. Typical current and charge times for xenon flasher unit as a function of input voltage.
